# Discovery and validation of genomic regions associated with resistance to maize lethal necrosis in four biparental populations

**DOI:** 10.1007/s11032-018-0829-7

**Published:** 2018-05-10

**Authors:** Manje Gowda, Yoseph Beyene, Dan Makumbi, Kassa Semagn, Michael S. Olsen, Jumbo M. Bright, Biswanath Das, Stephen Mugo, L. M. Suresh, Boddupalli M. Prasanna

**Affiliations:** 1 International Maize and Wheat Improvement Center (CIMMYT), P. O. Box 1041, Village Market, Nairobi, 00621 Kenya; 2 Department of Agricultural, Food and Nutritional Science, University of Alberta, Edmonton, Canada; 3 MRI-Syngenta, Lusaka, Zambia

**Keywords:** MLN, MCMV, QTL mapping, Joint linkage association mapping, Maize, GBS

## Abstract

In sub-Saharan Africa, maize is the key determinant of food security for smallholder farmers. The sudden outbreak of maize lethal necrosis (MLN) disease is seriously threatening the maize production in the region. Understanding the genetic basis of MLN resistance is crucial. In this study, we used four biparental populations applied linkage mapping and joint linkage mapping approaches to identify and validate the MLN resistance-associated genomic regions. All populations were genotyped with low to high density markers and phenotyped in multiple environments against MLN under artificial inoculation. Phenotypic variation for MLN resistance was significant and heritability was moderate to high in all four populations for both early and late stages of disease infection. Linkage mapping revealed three major quantitative trait loci (QTL) on chromosomes 3, 6, and 9 that were consistently detected in at least two of the four populations. Phenotypic variance explained by a single QTL in each population ranged from 3.9% in population 1 to 43.8% in population 2. Joint linkage association mapping across three populations with three biometric models together revealed 16 and 10 main effect QTL for MLN-early and MLN-late, respectively. The QTL identified on chromosomes 3, 5, 6, and 9 were consistent with the QTL identified by linkage mapping. Ridge regression best linear unbiased prediction with five-fold cross-validation revealed high accuracy for prediction across populations for both MLN-early and MLN-late. Overall, the study discovered and validated the presence of major effect QTL on chromosomes 3, 6, and 9 which can be potential candidates for marker-assisted breeding to improve the MLN resistance.

**Electronic supplementary material:**

The online version of this article (10.1007/s11032-018-0829-7) contains supplementary material, which is available to authorized users.

## Introduction

Maize is sub-Saharan Africa’s (SSA) most important staple food crop and is cultivated on more than 35 million hectares predominantly under rain-fed conditions and subject to the vagaries of weather (Shiferaw et al. 2011). The maize lethal necrosis (MLN) disease emerged as one of the major threats to the maize-based food security in SSA since 2011 (http://mln.cimmyt.org/). This devastating disease was first reported in September 2011 in the South Rift Valley of Kenya and by 2014, MLN was extensively reported in Kenya, Uganda, Tanzania, Rwanda, D.R. Congo, and Ethiopia (Wangai et al. 2012; Adams et al. 2014; Lukanda et al. 2014; Mahuku et al. 2015a, b). The MLN disease is caused by co-infection by two viruses—*Maize Chlorotic Mottle Virus* (MCMV) and *Sugarcane Mosaic Virus* (SCMV). Field observations indicated that MLN has affected almost all the commercially grown maize varieties in Kenya (De Groote et al. 2016), with yield losses ranging from 30 to 100% depending on the stage of disease infection and the environment.

Multi-mode transmission of both MLN-causing viruses is a significant challenge for effective management of the disease. SCMV is known to be transmitted by aphids in a non-persistent mode (Louie 1980; Tao et al. 2013) whereas MCMV is known to be transmitted by insect-vectors (including varied species of chrysomelid beetles and thrips) as well as contaminated seed (Jensen et al. 1991; Zhang et al. 2011). Application of chemical pesticides is resource-intensive for smallholders in eastern Africa and is not environment-friendly over a long term. Therefore, breeding for MLN resistance is a sustainable management option. The severity of MLN is widely influenced by favorable environments (Mahuku et al. 2015b). Screening germplasm under artificial inoculation against MLN is reliable, but is labor-intensive. Better understanding of the genetic basis of resistance to MLN can pave the way to accelerate the development of MLN-resistant germplasm.

Linkage mapping is commonly used to detect the quantitative trait loci (QTL) in biparental populations (Mackay et al. 2009). The QTL conferring resistance to SCMV and other major virus diseases in maize has been investigated in several studies particularly in temperate germplasm (Wisser et al. 2006; Redinbaugh and Pratt 2009; Ding et al. 2012; Zambrano et al. 2014; Li et al. 2016). Genome-wide association study (GWAS) on tropical maize germplasm showed that MLN is controlled by few loci with major effects and many loci with minor effects (Gowda et al. 2015). Joint linkage association mapping (JLAM) with multiple biparental populations offers an additional advantage over other mapping approaches by combining the high power of QTL detection from linkage analyses with the fine resolution of association mapping (Yu et al. 2008; Liu et al. 2011). However, the benefits of linkage mapping and JLAM have not been explored yet to understand the genetic architecture of MLN resistance.

In SSA, development and deployment of improved maize germplasm with enhanced yield and yield stability in disease-prone environments are the topmost priority (Cairns et al. 2013). Successful deployment of climate-resilient improved maize germplasm depends largely on improvement of relevant adaptive traits, including resistance to MLN, maize streak virus (MSV), Northern corn leaf blight (NCLB), gray leaf spot (GLS), and ear rots. Identifying and validating genomic regions conferring resistance to MLN and developing production markers can significantly accelerate the efforts on rapid development and deployment of elite, multiple stress-tolerant maize germplasm in SSA.

Genomic selection (GS) is rapidly gaining importance in plant breeding to accelerate genetic gain (Crossa et al. 2010, 2013, 2017; Vivek et al. 2017; Zhao et al. 2012; Zhang et al. 2017). Predicting and identifying the best resistant or best performing lines before phenotyping from the selected biparental populations is one of the most important applications of GS in maize breeding. Moderate to high accuracy has been reported in biparental populations of maize (Zhao et al. 2012; Zhang et al. 2015, 2017). In this study, our aim was to improve the understanding of the genetic architecture of MLN resistance in tropical maize germplasm, including identification/validation of genomic regions associated with MLN resistance. We applied linkage mapping, JLAM, and GS on four different biparental populations genotyped with low to high density markers and phenotyped in multiple environments in Kenya, under artificial inoculation with optimum combinations of MLN-causing viruses. The specific objectives were to (i) investigate the phenotypic variation for MLN resistance; (ii) identify/validate the genomic regions associated with MLN resistance by linkage mapping and JLAM; and (iii) evaluate the potential of GS for improving MLN resistance.

## Materials and methods

### Plant materials and field trials

Four biparental maize populations from the Global Maize Program of International Maize and Wheat Improvement Center (CIMMYT) were evaluated in MLN screening facility under artificial inoculation. Population 1 comprised of 229 F_3_ families from the cross, CML543 × LaPostaSeqC7-F71-1-2-1-2-B-B-B-B, population 2 comprised of 200 F_3_ families from the cross, CML543 × CML444, and *population 3* comprised of 260 F_3_ families from the cross, CML444 × CML539. In addition, population 4 comprised of 124 F_3_ families obtained from the cross, Mo37 × CML144 were also used in this study. All 689 F_3_ families from the first three populations were crossed with a common single-cross tester (CML312 × CML442) from the opposite heterotic pool, whereas population 4 was used as per se for phenotypic evaluation. The testcross progenies and F_3_ families of population 4 were evaluated in one row (3 m) plots with two replications in three seasons in two locations during 2012 to 2014 in Kenya. The locations were Narok (latitude 01° 05′ S, longitude 35° 52′ E, 1827 m above sea level (masl), clay loam texture) and Naivasha (latitude 0° 43′ S, longitude 36° 26′ E, 2086 masl, clay sandy loam soil texture). All standard agronomic management practices were followed. All lines were evaluated in replicated trials with α-lattice design.

### Artificial inoculation of MLN viruses

Stock isolates of MCMV and SCMV, collected from MLN hotspot areas in Kenya, were further confirmed by enzyme-linked immunosorbent assay (ELISA). In an MLN quarantine facility established in Naivasha, Kenya, both viruses were propagated on a susceptible hybrid, H614, in isolated greenhouses. Infected leaf samples collected from the field were cut into small pieces and ground in a mortar and pestle in extraction buffer (10 mM potassium–phosphate, pH 7.0). The resulting sap extract was centrifuged for 2 min at 12,000 rpm. Carborundum was added to decanted sap extract at the rate of 0.02 g/ml. The susceptible hybrid H614 at two leaves stage was inoculated by rubbing sap extract onto the leaves. These infected plants served as a source of inoculum for large-scale field trials. Two separate, sealed greenhouses were maintained for SCMV and MCMV inoculum production. Three weeks before harvesting the plants for field inoculation, random samples from the inoculated plants were tested with ELISA from the SCMV and MCMV greenhouses to confirm the inoculum purity.

Keeping the uniform disease pressure across field trials is crucial to get high-quality data. After several experiments, we optimized the optimum combination of SCMV and MCMV to have maximum MLN infection on maize plants. The ratio of 4 parts of SCMV and 1 part of MCMV (weight/weight) combination was more ideal (Gowda et al. 2015; Mahuku et al. 2015b). We used this optimized combination of SCMV and MCMV viruses (ratio of 4:1) and inoculated twice at the fourth and fifth week after planting. Plants were inoculated using a motorized, backpack mist blower (Solo 423 MistBlower, 12 ltr capacity). An open nozzle (2-in. diameter) was used to deliver inoculum spray at a pressure of 10 kg/cm^2^. Presence of both viruses in the field trials was confirmed by ELISA once disease symptoms were apparent (approximately 2 weeks post inoculation). MLN disease severity was visually scored on each plot in an ordinal scale of 1 (highly resistant, with no disease symptoms) to 5 (highly susceptible, leading to necrosis and death). Data were recorded twice as “early stage of infection” (21 days 1st post inoculation; hereafter referred to as “MLN-early”) and “late stage of infection” (42 days 1st post inoculation; hereafter referred to as “MLN-late”).

### Phenotypic evaluation

The testcross progenies from the first three populations (Pop1, Pop2, and Pop3) were evaluated in two seasons in Naivasha and one season in Narok, in separate but adjacent field trials connected with four common checks, whereas the fourth population (Pop4) was evaluated separately at two seasons in Naivasha. For the analyses, each season was treated as different locations. Observed outliers were excluded from analysis. Since MLN data were based on ordinal scales, it was evaluated to know whether the data meets the assumptions of the applied statistical model (independent, normally distributed, and constant variance; Rawlings et al. 1998). For each population, residuals plot and histogram across locations revealed that the MLN data meets all the model assumptions, and consequently, data was not transformed.

Analyses of variance for each of the location and across locations for each population were carried out using the PROC MIXED procedure with restricted maximum likelihood (REML) option in SAS 9.2 (SAS Institute 2010). Variance components were determined by following linear mixed model: *Y*_*ijko*_ = *μ* + *g*_*i*_ + *l*_*j*_ + *r*_*kj*_ + *b*_*ojk*_ + *e*_*ijko*_, where *Y*_*ijko*_ was the disease severity of the *i*th genotype at the *j*th environment in the *k*th replication of the *o*th incomplete block, *μ* was an intercept term, *g*_*i*_ was the genetic effect of the *i*th genotype, *l*_*j*_ was the effect of the *j*th environment, *r*_*kj*_ was the effect of the *k*th replication at the *j*th environment, *b*_*ojk*_ was the effect of the *o*th incomplete block in the *k*th replication at the *j*th environment, and *e*_*ijko*_ was the residual. Locations and replications were treated as fixed effects, and genotype and incomplete blocks as random effects. For JLAM, combined analyses of the first three populations were carried out to calculate best linear unbiased predictions (BLUPs) and total variance components by using MEATA-R software (http://hdl.handle.net/11529/10201). Heritability (*H*^2^) on an entry-mean basis was calculated as the ratio of genotypic to phenotypic variance.

### Molecular analyses

Six parental lines and their F_3_ progenies were genotyped with preselected, polymorphic, low-density SNPs by Monsanto Company, using a TaqMan assay (http://www.appliedbiosystems.com website), under the Water Efficient Maize for Africa (WEMA) project. In addition, the first three populations were also genotyped with high-density markers by genotyping-by-sequencing (GBS) at the Institute for Genomic Diversity, Cornell University, Ithaca, USA, as per the procedure described in earlier studies (Elshire et al. 2011; Glaubitz et al. 2014; Gowda et al. 2015). The detailed information on low-density markers were described in previous study (Semagn et al. 2013).

### Linkage mapping

For the first three populations (Pop1, Pop2, and Pop3), the GBS data was filtered with a minor allele frequency (MAF) of 0.05 and a minimum count of 95% of the sample size. Then, only marker loci homozygous for both parents and polymorphic between the two parents were retained in all populations. After quality screening, set of uniformly distributed, polymorphic SNPs was selected. For each marker locus, observed genotype frequencies were checked for deviations from Mendelian segregation ratios and allele frequency of 0.5 using a *χ*^2^ test. High-quality molecular data were used to construct genetic linkage maps. For population 4, a linkage map was constructed using low-density markers. Individual linkage maps for each population were constructed by using QTL IciMapping software ver 4.0 (Meng et al. 2015; http://​www.​isbreeding.​net). Linkage analyses of SNPs were conducted using the Kosambi (1944) mapping function with a minimum logarithm of odds (LOD) of 3.0 and a maximum distance of 30 cM between two loci.

For each population, BLUPs across locations for MLN-early and MLN-late disease scores were used to detect QTL based on inclusive composite interval mapping (ICIM) implemented in the software QTL IciMapping V.4 (Meng et al. 2015). With the ICIM method, the walking step in QTL scanning of 1 cM and a relaxed LOD threshold of 3.0 were chosen for declaring putative QTL. The origin of the favorable alleles for MLN resistance was identified based on sign of the additive effects of each QTL.

### Joint linkage association mapping

For JLAM, GBS-based SNPs from the first three populations were used. For quality screening, in each population, SNPs which were either monomorphic between the parents, or had missing value of > 5%, or had a minor allele frequency of < 0.05 were discarded from analysis. After these quality checks, 15,000 high-quality GBS SNPs were retained for JLAM analyses across populations. BLUPs calculated across populations and environments were used in JLAM studies.

For the JLAM approaches, an additive genetic model was chosen for the testcross progenies (Utz et al. 2000). We used three multiple regression approaches for JLAM and each of these models was explained in detail by Liu et al. (2011) and Würschum et al. (2012). In brief, we applied a two-step procedure for QTL detection. In a first step, stepwise multiple linear regression was used to select a cofactors based on the Schwarz Bayesian Criterion (SBC, Schwarz 1978). Cofactors were selected by using Proc GLM SELECT implemented in the statistical software SAS 9.2 (SAS Institute 2010). In the second step, we calculated a *P* value for the *F* test by using a full model (including SNP effect) versus a reduced model (without SNP effect) (Reif et al. 2010). Genome-wide scans for QTL were implemented in R version 3.2.5 (R Development Core Team 2013).

The first model, model A, for QTL detection is as follows: Trait *= Cofactors* + Marker, it includes both cofactors and marker effects across populations (for details, see Reif et al. 2010; Liu et al. 2011). In model B, a population effect is included as additional effect to correct the population stratification: Trait *= Pop* + *Cofactors* + Marker. In the third model (model C), both cofactors and marker effects were modeled as nested within populations: Trait *= Pop* + *Cofactors* (*Pop*) + Marker (*Pop*). Bonferroni–Holm procedure (Holm 1979) was used to declare markers significantly (*P* < 0.05) associated with MLN disease resistance. The total proportion of phenotypic variance explained by the detected QTL was calculated by fitting all significant SNPs simultaneously in a linear model to obtain adjusted *R*^2^ (Utz et al. 2000). Principal components (PC) were calculated using TASSEL ver 5.0 (Bradbury et al. 2007).

The physical positions of all the markers mapped in all four populations are known. We developed integrated physical map by including all the markers from four populations. All the QTL detected for MLN-early and MLN-late in each populations and JLAM were mapped on this integrated physical map. The 60-bp source sequences of the significant SNP were used to perform BLAST searches against the ‘B73’ RefGen_v2 (http://blast.maizegdb.org/home.php?a=BLAST_UI). Within the local LD block including associated SNPs, the filtered genes in MaizeGDB (http://www.maizegdb.org) containing directly or adjacent to each significant SNP were considered as possible candidate genes for MLN resistance.

### Genome-wide prediction

For genome-wide prediction, 2000 common SNPs for each of the three populations which had no missing values and distributed uniformly across the genome were selected. For GS, the ridge regression best linear unbiased prediction (RR-BLUP; Whittaker et al. 2000) method was used (Zhao et al. 2012). Prediction accuracy of the GS approach was evaluated using five-fold cross-validation with 100 times repetitions. The accuracy of GS was calculated as *r*_*GS*_ = *r*_*MP*_/h, where *h* refers to the square root of heritability and *r*_*MP*_ is the correlation between observed and predicted phenotypes (Dekkers 2007). The prediction accuracies for MLN resistance were compared based on random markers and combination of random markers with significant markers detected through JLAM.

Further, to understand the effect of different training populations on prediction accuracy, GS was applied to predict within and across biparental populations. We estimated the marker effect and predicted the genomic breeding values in two different scenarios as follows: scenario 1a: Estimation of marker effects was performed across populations, and prediction accuracy was assessed across populations; scenario 1b: Estimation of marker effects was performed across populations, and prediction set was drawn from within each population. In scenario 2, estimation of marker effects and prediction of genomic breeding values were performed within each segregating population. For scenario 1a and b, estimation of marker effects was based on the genotypic variance of the total populations. In contrast, scenario 2 was based on the estimates of the average genotypic variance and heritability within segregating populations.

## Results

Among the six parental lines used in this study CML543, CML539, and CML144 are moderately resistant or tolerant with mean score of 2.1, 2.2, and 2.1 for MLN-early and 2.3, 2.5, and 2.4, for MLN-late, respectively. Whereas, CML444, LaPostaSeqC7-F7,1 and Mo37 are susceptible to MLN with the mean score of 2.8, 3.1, and 3.3 for MLN-early, and 3.4, 3.6, and 4.1, for MLN-late, respectively. We observed a wide variation for MLN disease severity at both early and late stages of infection (Fig. S1). The analyses across environments revealed significant (*P* < 0.01) variances for genotypes, and environments, for both MLN-early and MLN-late in all four populations (Table 1). Genotype × environment interaction variances were significant for first two populations for both MLN-early and MLN-late. Genotypic variances among populations (σ^2^_G-Among_ = 0.021 and 0.042 for MLN-early and MLN-late, respectively) were of the same magnitude as those of within populations (σ^2^_G-Within_ = 0.020 and 0.023 for MLN-early and MLN-late, respectively). The estimates of broad-sense heritability were moderate to high ranging from 0.34 to 0.83 for MLN-early and 0.44 to 0.89 for MLN-late scores. Consequently, phenotyping in multiple locations under artificial inoculations resulted in high-quality data representing an excellent resource to study the genetic architecture of MLN disease resistance.

**Table 1:** Analysis of variance components for MLN disease severity evaluated across two to three environments with four different F_3_ mapping populations

Trait	*σ* ^2^_G_	*σ* ^2^_GE_	*σ* ^2^_e_	*h* ^2^
CML543 × LaPostaSeqC7-F71				
MLN-early	0.05*	0.02*	0.17	0.58
MLN-late	0.06*	0.06*	0.23	0.52
CML543 × CML444				
MLN-early	0.04*	0.01*	0.18	0.53
MLN-late	0.07*	0.02*	0.24	0.60
CML539 × CML444				
MLN-early	0.02*	0.01	0.21	0.34
MLN-late	0.03*	0.02*	0.20	0.44
M037 × CML144				
MLN-early	0.08*	0.001	0.09	0.83
MLN-late	0.21*	0.001	0.15	0.89
Across three pops				
MLN-early	0.03*	0.002*	0.19	0.25
MLN-late	0.04*	0.03	0.15	0.35

*Significance at < 0.05 level of probability

Linkage maps were constructed for all four F_3_ populations. The number of progenies, SNPs, map length, and average genetic distance between SNPs for each population are presented in supplementary Table S1. The QTL mapping results revealed that the number of QTL associated with MLN-early varied from 2 to 8, with total phenotypic variance explained ranging from 38.8% in population 4 to 56.8% in population 2 (Table 2). In contrast, the number of QTL associated with resistance to MLN at late stage (MLN-late) varied from 3 to 6, with total phenotypic variance explained ranging from 37 to 58.6%. One QTL each in population 1 and population 4, three QTL in population 2, and two QTL on population 3 were consistently detected for both MLN-early and MLN-late data. The proportion of phenotypic variance explained by single QTL in each population ranged from 3.9 to 37.8% for population 1, 4.2 to 43.8% for population 2, 4.8 to 14.1% for population 3, and 9.6 to 16.6% for population 4 (Table 2). For MLN-early, three major QTL located on chromosomes 3, 6, and 9 were consistently detected in at least two populations. For MLN-late, one QTL each located on chromosomes 3 and 6 was consistently expressed in two populations (Table 2).

**Table 2:** Detection of QTL associated with resistance to MLN at early and late stages of disease infection, and their physical positions and genetic effects of the QTL in four F_3_ populations

Trait	QTL name^a^	Chr	Position (cM)	LOD	PVE (%)	Add	Dom	PVE (%)	QTL confidence interval		Physical position (bp)		Fav parent
									Left marker	Right marker	Left	Right	
CML543 × LaPostaSeqC7-F71													
MLN-early	*qMLN_03-01*	3	0	3.80	3.96	− 0.09	0.17	48.22	S3_1326446	S3_1337663	1326446	1337663	LPS_F71
	*qMLN_03-130*	3	157	27.48	37.80	0.27	0.00		*PZA00413_20*	*PZA02299_16*	*125192432*	*130082791*	CML543
	*qMLN_06-04*	6	5	5.12	5.61	0.08	0.02		S6_3869709	S6_4015237	3869709	4015237	CML543
MLN-late	*qMLN_01-252*	1	68	3.09	3.41	− 0.08	0.00	48.23	S1_251157170	PZA02269_4	251157170	252721946	LPS_F71
	*qMLN_03-130*	3	157	21.37	27.16	0.29	− 0.03		*PZA00413_20*	*PZA02299_16*	*125192432*	*130082791*	CML543
	*qMLN_06-19*	6	46	8.52	9.40	0.14	− 0.06		S6_18924381	S6_21021616	18924381	21021616	CML543
	*qMLN_08-174*	8	222	5.06	5.45	− 0.10	0.03		S8_174216280	S8_174222902	174216280	174222902	LPS_F71
CML444 × CML543													
MLN-early	*qMLN_01-246*	1	304	4.57	4.99	0.07	− 0.02	56.75	*S1_246489667*	*S1_246540548*	*246489667*	*246540548*	CML543
	*qMLN_03-146*	3	123	30.07	43.84	0.25	0.01		*S3_146251234*	*S3_146250249*	*146251234*	*146250249*	CML543
	*qMLN_06-85*	6	197	5.73	6.12	0.08	0.01		*S6_85416016*	*S6_96909472*	*85416016*	*96909472*	CML543
	*qMLN_08-123*	8	94	3.81	4.23	0.02	− 0.11		S8_123055373	S8_123469828	123055373	123469828	CML543
	*qMLN_10-81*	10	213	4.22	4.43	− 0.07	0.00		S10_81827920	S10_81829237	81827920	81829237	CML444
MLN-late	*qMLN_01-241*	1	282	7.02	10.46	0.00	− 0.24	58.61	*S1_237487786*	*S1_241184216*	*237487786*	*241184216*	CML543
	*qMLN_03-146*	3	124	20.08	24.26	0.26	− 0.03		*S3_146250249*	*S3_146251923*	*146250249*	*146251923*	CML543
	*qMLN_05-190*	5	251	9.55	10.44	− 0.16	0.01		S5_190677275	PZA00352_23	190677275	191075557	CML444
	*qMLN_05-199*	5	349	15.36	17.74	0.19	0.00		S5_199499548	S5_199499538	199499548	199499538	CML543
	*qMLN_05-207*	5	432	4.06	4.25	− 0.04	0.13		S5_206890892	PZA02015_11	206890892	207464707	CML444
	*qMLN_06-85*	6	176	10.32	10.90	0.15	0.06		*S6_85203511*	*S6_85206463*	*85203511*	*85206463*	CML543
CML539 × CML444													
MLN-early	*qMLN_01-148*	1	72	3.24	4.84	− 0.07	0.01	42.15	ZM00148950	ZM00145696	148500000	160300000	CML539
	*qMLN_03-27*	3	69	4.17	6.91	0.02	− 0.11		S3_27365043	S3_29611811	27365043	29611811	CML444
	*qMLN_03-126*	3	350	6.32	10.55	0.00	0.14		S3_122493752	S3_126171099	122493752	126171099	CML444
	*qMLN_04-117*	4	59	5.54	9.13	0.09	− 0.04		ZM00148564	ZM00146885	106500000	117900000	CML444
	*qMLN_05-131*	5	150	4.33	6.46	0.08	0.02		ZM00147053	ZM00148930	115900000	131900000	CML444
	*qMLN_06-03*	6	185	5.42	8.96	− 0.05	− 0.11		*S6_2385933*	*S6_3039514*	*2385933*	*3039514*	CML539
	*qMLN_09-100*	9	66	4.37	7.49	− 0.08	− 0.02		*S9_99440296*	*S9_100608734*	*99440296*	*100608734*	CML539
	*qMLN_10-71*	10	32	5.42	8.21	0.09	0.01		S10_71178058	S10_71665925	71178058	71665925	CML444
MLN-late	*qMLN_03-159*	3	487	5.48	9.13	− 0.10	0.03	36.95	S3_159801859	S3_158524074	159801859	158524074	CML539
	*qMLN_05-171*	5	167	3.79	6.16	0.09	− 0.03		ZM00147844	ZM00150817	154700000	171900000	CML444
	*qMLN_06-06*	6	202	8.15	14.15	− 0.13	− 0.01		*S6_5159730*	*S6_6270908*	*5159730*	*6270908*	CML539
	*qMLN_06-16*	6	215	5.68	9.51	0.00	− 0.16		ZM00150684	S6_16291106	12700000	16291106	CML444
	*qMLN_09-100*	9	230	3.51	7.27	0.01	− 0.13		*ZM00149322*	*ZM00145937*	*96500000*	*116800000*	CML444
Mo37 × CML144													
MLN-early	*qMLN_03-130*	3	129.6	5.6	16.63	0.11	0.03	38.76	*PHM1745_16*	*PZA00920_1*	*129095914*	*131969810*	CML144
	*qMLN_09-100*	9	112.5	5.33	16.32	− 0.05	− 0.43		PZA00060_2	PZB00221_3	95769540	113201792	Mo37
MLN-late	*qMLN_03-129*	3	128.6	5.58	13.97	0.18	− 0.04	39.13	*PZA00413_20*	*PHM1745_16*	*113820730*	*129095914*	CML144
	*qMLN_06-19*	6	14.2	3.18	9.61	− 0.14	− 0.02		PZA00158_2	PZA00440_1	13254840	21599925	Mo37
	*qMLN_08-157*	8	156.4	4.32	13.94	− 0.15	0.12		PZA00460_3	PHM4786_9	156320002	157402090	Mo37

MLN-early indicates MLN score 21 days after first post inoculation; MLN-late indicates MLN score 42 days after first post inoculation; fav parent indicates parental genotype from where favorable allele for MLN resistance is contributing. Markers with italicized letters are the QTL consistent across MLN-early and MLN-late

*LOD* logarithm of odds, *Add* additive effect, *Dom* dominance effect, *PVE* phenotypic variance explained

^a^QTL name composed by the trait code followed by the chromosome number in which the QTL was mapped and a physical position of the QTL

The first two principal components explained 24% of the total variation (Fig. S2). The PCA revealed a population structure of the four parents with three clusters. JLAM analyses with three biometric models together revealed 16 and 10 main effect QTL for MLN-early and MLN-late, respectively (Table 3). With model A, six QTL each were associated with MLN-early and MLN-late and were identified, which together explained 38.27 and 16.44% of the total phenotypic variance, respectively. With model B, by including population effect, nine QTL were detected for MLN-early whereas only one QTL was detected for MLN-late. Model C by considering the nested effect of SNPs in each population, three and four different QTL were identified for MLN-early and MLN-late, respectively. Across these models, one QTL each was overlapped between models A and B and models A and C for MLN-early, whereas only one overlapped QTL was detected between models A and B for MLN-late.

**Table 3:** Analysis of trait-associated markers, allele substitution (α) effects, and the total phenotypic variance (*R*^2^) of the joint linkage association mapping in multiple segregating F_3_ populations based on three different biometrical models

MLN_early	QTL name^a^	chr	Position (Mbp)	Model A			Model B			Model C		
				α-Effect	*P* value	PVE (%)	α-Effect	*P* value	PVE (%)	α-Effect	*P* value	PVE (%)
S1_35581569	*qMLN_01-36*	1	35.58	–	–	–	0.21	1.18E−13	7.50	–	–	–
S1_293055726	*qMLN_01-293*	1	293.06	0.12	1.85E−10	5.90	–	–	–	–	–	–
S3_56468658	*qMLN_03-56*	3	56.46	0.21	1.08E−20	21.70	0.27	1.38E−38	21.70	–	–	–
S3_138773656	*qMLN_03-139*	3	138.77	− 0.15	5.64E−22	8.50	–	–	–	− 0.17	5.64E−22	19.90
S4_5150195	*qMLN_04-05*	4	5.15	0.11	8.72E−09	2.50	–	–	–	–	–	–
S6_21886770	*qMLN_06-21*	6	21.88	0.20	2.30E−18	10.10	–	–	–	–	–	–
S6_39371783	*qMLN_06-39*	6	39.37	–	–	–	–	–	–	− 0.06	1.57E−07	3.50
S6_82022555	*qMLN_06-82*	6	82.02	–	–	–	0.12	7.88E−07	2.70	–	–	–
S6_99946471	*qMLN_06-100*	6	99.94	–	–	–	− 0.08	2.00E−07	3.00	–	–	–
S6_120159068	*qMLN_06-120*	6	120.16	–	–	–	0.11	7.36E−07	2.70	–	–	–
S6_158478115	*qMLN_06-158*	6	158.48	–	–	–	0.10	1.48E−07	1.60	–	–	–
S7_3671560	*qMLN_07-037*	7	3.67	0.08	1.57E−07	3.20	–	–	–	–	–	–
S8_147097693	*qMLN_08-147*	8	147.09	–	–	–	0.14	1.13E−10	4.70	–	–	–
S9_94515942	*qMLN_09-95*	9	94.51	–	–	–	–	–	–	0.07	8.45E−07	2.70
S9_137154420	*qMLN_09-137*	9	137.15	–	–	–	0.08	8.45E−07	3.10	–	–	–
S10_145280961	*qMLN_10-145*	10	145.28	–	–	–	0.11	1.47E−11	6.00	–	–	–
Total PVE (%)						38.27			30.81			25.5
MLN-late												
S1_7162859	*qMLN_01-071*	1	7.16	− 0.13	1.34E−06	2.80	–	–	–	–	–	–
S2_30361545	*qMLN_02-30*	2	30.36	0.16	8.08E−09	2.90	0.16	6.14E−08	4.20	–	–	–
S3_119323182	*qMLN_03-119*	3	119.32	− 0.13	9.58E−08	3.60	–	–	–	–	–	–
S3_133187288	*qMLN_03-133*	3	133.19	–	–	–	–	–	–	− 0.11	2.30E−18	15.00
S3_188926823	*qMLN_03-189*	3	188.93	0.11	1.29E−09	4.80	–	–	–	–	–	–
S5_205155852	*qMLN_05-205*	5	205.16	–	–	–	–	–	–	0.01	9.58E−08	3.60
S6_5441847	*qMLN_06-05*	6	5.44	–	–	–	–	–	–	− 0.02	1.29E−09	4.80
S6_38273901	*qMLN_06-39*	6	38.27	0.09	3.52E−07	4.30	–	–	–	–	–	–
S7_19623847	*qMLN_07-19*	7	19.62	0.15	1.23E−10	5.60	–	–	–	–	–	–
S7_123880597	*qMLN_07-123*	7	123.88	–	–	–	–	–	–	0.09	3.52E−07	4.10
Total PVE (%)						16.44			4.19			7.91

MLN-early indicates MLN score 21 days after first post inoculation; MLN-late indicates MLN score 42 days after first post inoculation; *R*^2^ indicates proportion of phenotypic variance explained

*Chr* chromosome, *MLM* mixed linear model, *MAF* minor allele frequency

^a^QTL name composed by the trait code followed by the chromosome number in which the QTL was mapped and a physical position of the QTL

JLAM is expected to increase the resolution within QTL intervals detected by individual population linkage analyses. Therefore, in this study, we tried to identify the QTL that fell within the confidence interval of MLN resistance QTL identified through the biparental approach. On chromosome 3, JLAM identified five SNP markers associated with MLN resistance QTL, among them, one SNP (S3_119323182) fell within the confidence interval of QTL identified in population 4 (113 to 129 Mbp) (Tables 2 and 3) whereas the same SNP fell just outside the confidence intervals of the major QTL in other three populations. SNP marker detected on chromosome 5 (S5_205155852) was located just outside the confidence interval of QTL detected on population 2. On chromosome 6, JLAM detected eight MLN resistance QTL. One of them located in bin 6.00 (S6_5441847) resided within the confidence interval of QTL identified in the population 3 (S6_5159730-S6_6270908). Other SNPs fell just outside the confidence interval of the QTL detected on chromosome 6 (Tables 2 and 3). On the chromosome 9, JLAM discovered one QTL that resided in bin 9.03 fell under the QTL detected on populations 3 and 4 (95.7 to 116.8 Mbp) (Tables 2 and 3). This SNP marker (S9_94515942) can serve as an anchor landmark to delimit the confidence interval for these QTL. A set of putative candidate genes associated with significant SNPs/QTL was identified (Table S2). All the QTL detected for individual populations and JLAM were mapped on one integrated map (Fig. S3).

We used five-fold cross-validation to assess the accuracy of genomic predictions for resistance to MLN-early and MLN-late traits by combining the data from the first three populations and within each population (Fig. 2a, b). The cross-validated prediction accuracy when both the training and estimation sets were formed across populations was high with 0.65 and 0.77 for MLN-early and MLN-late, respectively. For both MLN-early and MLN-late, the prediction accuracy was slightly improved by 2% with inclusion of the MLN resistance-associated markers into the prediction model (Fig. 2a). When the training set was derived from across populations and the prediction set was within population, the prediction accuracy was high and varied from 0.58 to 0.72 for MLN-early and 0.65 to 0.73 for MLN-late. Prediction accuracy of genomic breeding values within each biparental population ranged from 0.71 to 0.76 for MLN-early and 0.68 to 0.82 for MLN-late scores (Fig. 2b).

## Discussion

MLN is a complex challenge that has to be effectively addressed through several simultaneously implemented strategies, including development and deployment of MLN-resistant germplasm (Prasanna 2016). Over the last 4 years, CIMMYT has screened more than 120,000 germplasm entries against MLN under artificial inoculation at the centralized MLN screening facility established in Naivasha, Kenya (http://mln.cimmyt.org/). Although a substantial proportion of pre-commercial and commercial maize germplasm in SSA is susceptible to MLN, these intensive efforts enabled identification of promising CIMMYT maize germplasm with tolerance/resistance to MLN, including the individual viruses (MCMV and SCMV).

Phenotype-based selection strategies are often resource-intensive and time-consuming. Identifying and validating MLN resistance-associated molecular markers which are stable across diverse genetic backgrounds could potentially enable pre-selection of genomic regions in Africa-adapted sub-tropical maize germplasm, thereby contributing to enhanced genetic gains. In this study, we performed linkage mapping, JLAM, and GS to understand the genetic architecture of MLN resistance and validate earlier findings in sub-tropical maize germplasm.

QTL analyses in each of the four populations identified three major QTL genomic regions on chromosome 3, between 113 and 131 Mbp (bin 3.04) and 145 and 160 Mbp (bin 3.05). Major QTL detected on chromosome 6 are also mapped in three genomic regions, between 2 and 6 Mbp, 15 and 21 Mbp, and 85 and 96 Mbp (bin 6.00/01). On chromosome 9, we found major consistent QTL on bin 9.03, between 95 and 116 Mbp. Interestingly, the major QTL on chromosome 3 consistently expressed at both early and late stages of MLN infection; however, most of the QTL on chromosome 6 showed stage-specific expression. Genomic regions particularly on bin 3.04 and bin 6.00/01 in chromosomes 3 and 6, respectively, are known as the regions rich in resistant genes to multiple maize viruses, including SCMV, *Maize dwarf mosaic virus* (MDMV), and *Johnson grass mosaic virus* (JGMV; Xia et al. 1999; Jones et al. 2007; Ingvardsen et al. 2010; Ding et al. 2012; Stewart et al. 2013; Tao et al. 2013; Zambrano et al. 2014), *Wheat streak mosaic virus* (WSMV; Jones et al. 2011), *Maize mosaic virus* (MMV; Zambrano et al. 2014), and *maize chlorotic dwarf virus* (MCDV; Jones et al. 2004; Zambrano et al. 2014). The results of the current study also indicate the importance of the same regions having QTL with major effects. Nevertheless, whether the same region/s are contributing for resistance to both SCMV and MCMV or SCMV alone warrants further study.

The major effect QTL on chromosomes 3, 6, and 9 are interesting targets for either marker-assisted backcrossing (MABC) or marker-assisted recurrent selection (MARS) to introgress into different genetic backgrounds particularly on highly susceptible, widely using elite lines. These QTL can also be potentially used in maize breeding with the aim to enrich target alleles in F2 populations prior to producing DH lines from such populations. In this study, we also found a few new major QTL on chromosomes 1, 5, and 8; however, these QTL were expressed in specific populations and at specific stages of MLN infection.

**Fig. 1:**
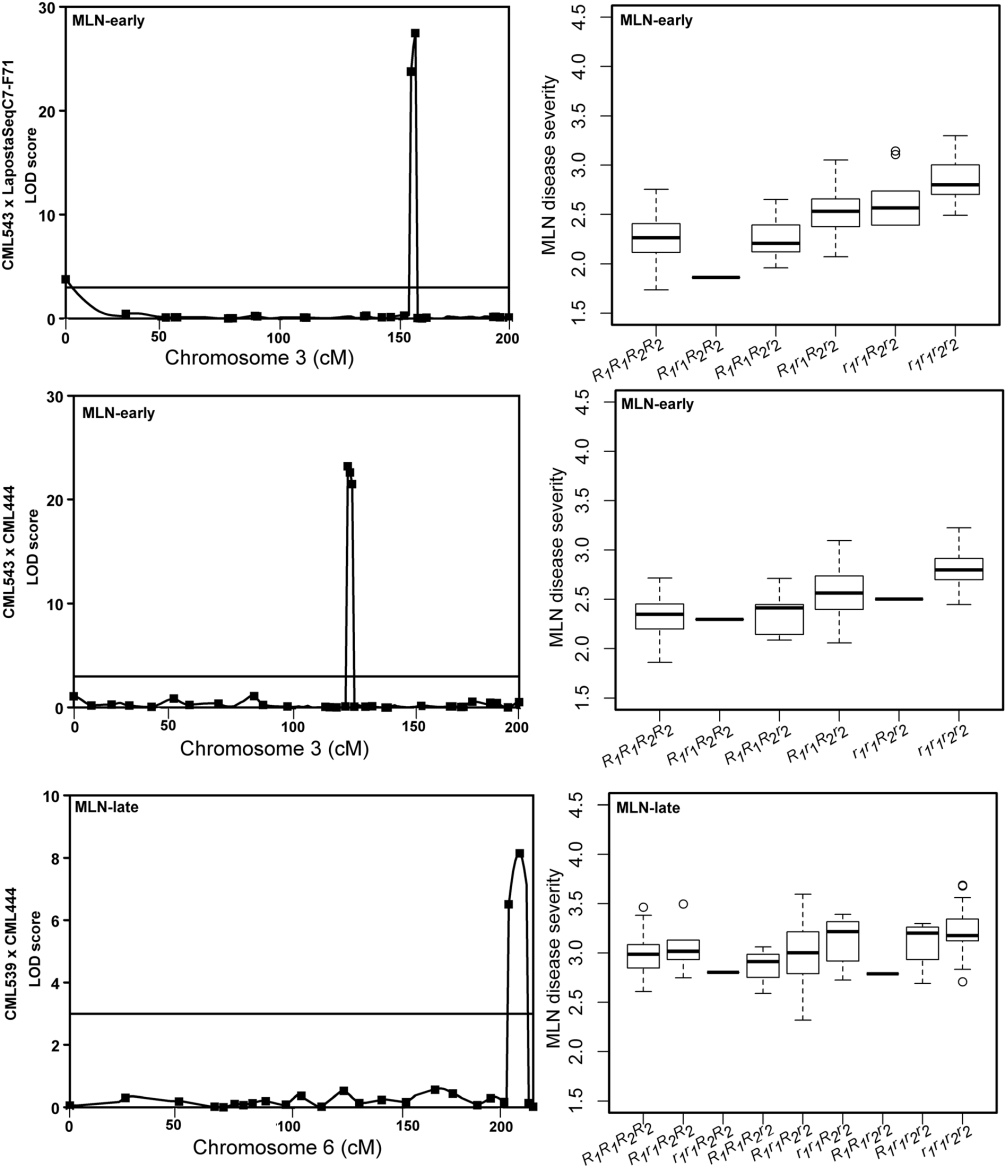
Major QTL for MLN resistance in three F_3_ populations. A likelihood of odds (LOD) scan showing the QTL identified on chromosomes 3 and 6 in three F_3_ populations. Box-whisker plots display the level of disease resistance or severity for different allele combinations at resistance gene loci explaining > 10% of the phenotypic variation for MLN-early and MLN-late as determined by two strongly associated SNP markers

In population 1, major QTL identified on chromosome 3 with a LOD score of 27.48 and explaining 37% of the phenotypic variation revealed that CML 543 is the source of favorable alleles. The segregation alleles from two tightly linked markers for this major QTL reveal that F3 plants with low disease severity score were strongly associated with alleles from CML543, the resistant parent (Fig. 1) for both MLN-early and MLN-late. Similar association was also found for other major QTL observed on population 2, with strong association between alleles from two closely linked markers with low disease severity data (Fig. 1). This suggests CML 543 can be used as a potential donor to introgress the major QTL identified on chromosome 3. In contrast, the distribution of MLN-late disease severity in population 3 with markers linked to QTL on chromosome 6 showed some differences in the action of genes for controlling MLN tolerance. The population 3 phenotypes for MLN-late were skewed toward the susceptible parent. F3 plants having homozygous dominant alleles from one marker locus and homozygous recessive allele from other marker loci showed strong association with low disease severity score. This warrants further study to clarify on whether the identified QTL on chromosome 6 carries one gene or more than one gene before concluding on the type of gene/s involved in MLN resistance (Fig. 1).

JLAM was implemented with the aim to take the advantages of both the high detection power of linkage mapping and improved resolution of association mapping to robustly identify the MLN resistance QTL. We applied three biometric models to increase the possibility to capture maximum number of QTL associated with MLN resistance. In this study, with three biometric models, we found 16 and 10 main effect QTL significantly associated with resistance to MLN at early and late stages of disease infection, respectively. The QTL identified on chromosomes 3, 5, 6, and 9 were consistent with the QTL found by single-population-based linkage analyses. Further, JLAM also drastically increased the resolution within the confidence intervals in some of MLN resistance QTL on chromosomes 3, 5, 6, and 9 (Tables 2 and 3). Moreover, we also found a few new QTL associated with MLN resistance that were not detected by linkage mapping but found only with JLAM. This could be attributed to the higher power and resolution offered by combined linkage and association mapping by exploiting both the variations across and within populations.

The results obtained in this study revealed some common genetic loci with previous large GWAS on MLN resistance (Gowda et al. 2015) and SCMV resistance (Zambrano et al. 2014; Li et al. 2016). QTL on chromosome 3 at 113, 133, and 189 Mbp were identified in both the association panel and in population 4 and JLAM panel. Similarly, QTL reported on chromosomes 5 (199 Mbp) and 6 (85 Mbp) in the association panel were also found in population 2 and population 3. Taken together, these results indicate that there is common genomic regions particularly across populations on chromosomes 3, 6, and 9 which contributing significantly on resistance to MLN.

**Fig. 2:**
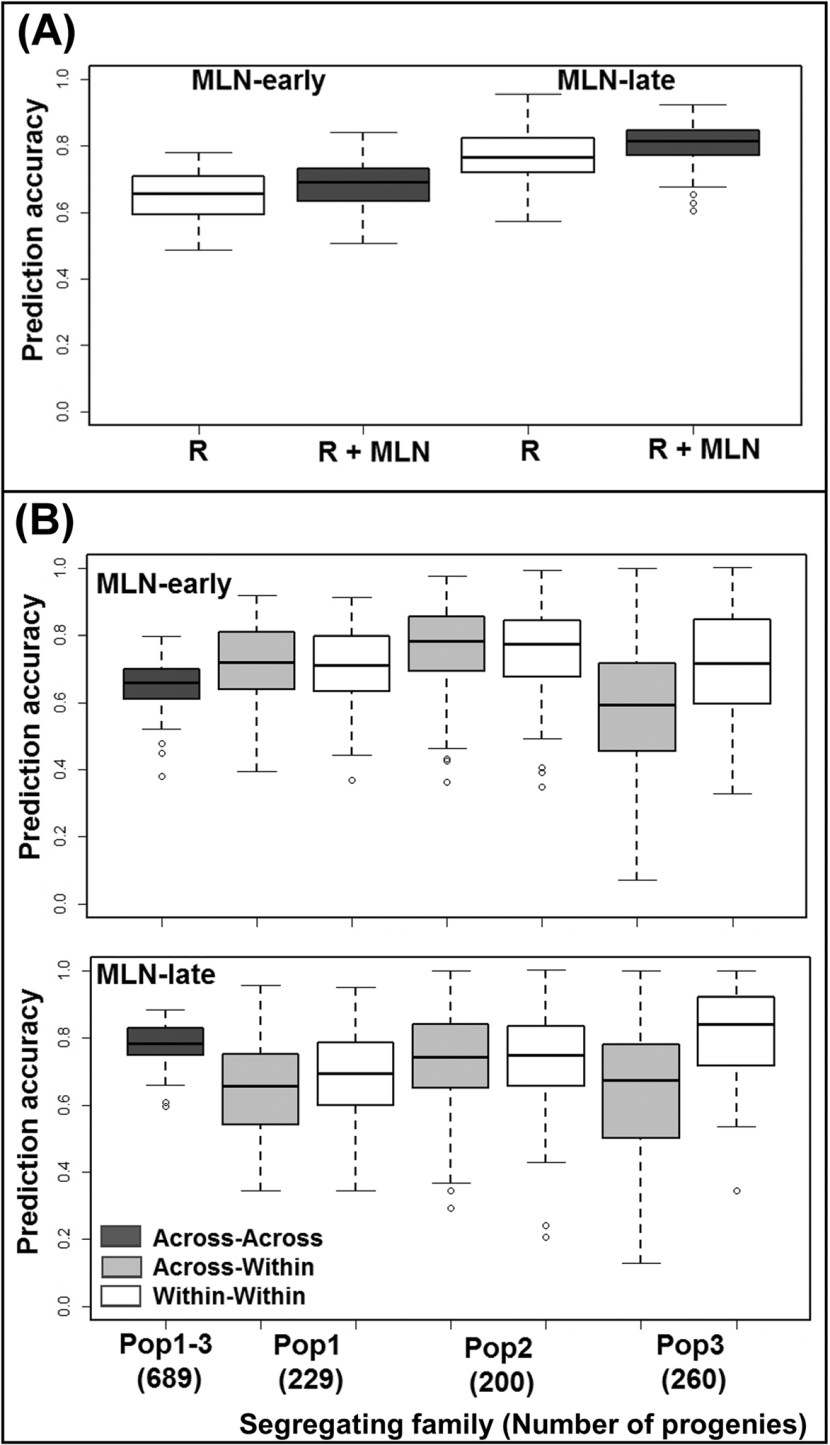
Genome-wide prediction accuracies based on random markers (**a**) (R) and random + MLN resistance associated significant markers (R + MLN), and prediction accuracy based on three different scenarios (**b**). Scenario 1a—estimation and prediction across families; scenario 1b—estimation across and prediction within families; and scenario 2—both estimation and prediction within biparental segregating families, with five-fold cross-validation for MLN disease severity at early and late stages of infection

The ability to predict and select best disease-resistant lines without phenotyping in biparental populations based on genomic-estimated breeding values is an important application of GS in maize breeding (Zhang et al. 2017). The primary method for doing this is through GS models (Meuwissen et al. 2001; Lorenz et al. 2011), a strategy that is well established in large commercial seed companies but still in its infancy among public sector breeding programs. The potential and limits of GS-based predictions have been examined in maize for several traits (Albrecht et al. 2011; Riedelsheimer et al. 2012; Zhao et al. 2012; Bernardo 2014; Crossa et al. 2017). Although MLN resistance is relatively complex (because of combination of two viruses), we observed high prediction accuracy of > 0.65 across three populations, which is comparable with the previously reported prediction accuracy for MLN (Gowda et al. 2015) and NCLB (Technow et al. 2013) (Fig. 2a). We observed small improvements in prediction accuracy by including MLN resistance-associated markers suggesting the possibility of considerable contribution from several minor effect QTL which were not detected by linkage mapping studies.

Success of GS in maize breeding depends on the type of training populations used and their genetic relationships with the prediction set. In this study, we tested predictions within and across biparental populations. The results clearly suggested that for MLN, the accuracy is not affected significantly when training populations are based on either a single biparental population or multiple populations or even when a selected panel of breeding lines (Gowda et al. 2015) is used. QTL mapping and JLAM results suggest that the genetic architecture of MLN is perhaps much less complex compared to traits like grain yield. Therefore, for comprehensive improvement of MLN resistance in breeding materials, we suggest to incorporate GS in breeding programs, as GS allows to capture contributions of even small effect QTL along with the major effect QTL. The prediction accuracy for MLN was slightly higher when the training set was derived from the same population than from a combination of many populations. This might be attributed to varying levels of relatedness and confounding population structure. Nevertheless, the prediction accuracy is still promising and encouraging to apply GS as one option to select the best MLN-resistant lines by reducing the phenotyping efforts.

High-throughput and cost-effective genotyping platforms are required to implement GS routinely in the breeding programs. Recent advances in sequencing technologies like GBS provide the capacity to genotype substantial number of breeding lines at low costs (Elshire et al. 2011). The cost per sample for GBS is comparable with the low-density SNPs obtained from the single-plex arrays. In this study, for across populations, phenotypic selection accuracy which is estimated as *h* (square root of heritability) was moderate for early and late stages of MLN infection. Whereas for GS, selection accuracy was slightly higher for MLN-early and MLN-late. By considering the possibility to complete up to three maize cycles per year (Lorenzana and Bernardo 2009), GS is more efficient in terms of genetic gain per year. With rapid reduction in genotyping cost, it is possible to effectively apply GS for MLN resistance routinely in maize breeding programs in SSA.

## Conclusion

In this study, we used four biparental populations to understand the genetic architecture of MLN resistance and validate the earlier findings in CIMMYT-derived sub-tropical maize germplasm. Two major QTL were identified on chromosomes 3 and 6 across different genetic backgrounds; these could be potential candidates for genomic-assisted breeding. JLAM scan also identified 26 main effect QTL significantly associated with resistance to MLN. The genomic regions identified on chromosomes 1, 3, 6, and 9 are consistently detected in both linkage mapping and JLAM. Further validation could lead to development of production markers for MLN resistance. Introgressing these major QTL on chromosomes 3, 6, and 9 into elite inbred lines could improve the level of resistance to MLN. GS results revealed higher genetic gain per year for marker-based selection. These results suggest that integration of GS in maize breeding even with small training population sizes is an attractive complement to phenotypic selection to improve resistance to MLN. Overall, the study confirmed that MLN resistance is controlled by a few major genes and several minor genes.

## Electronic supplementary material

ESM 1 (DOCX 101 kb)

ESM 2 (DOCX 137 kb)

ESM 3 (DOCX 1.73 mb)

ESM 4 (DOCX 12.6 kb)

ESM 5 (DOCX 17.4 kb)
