# Supplementary material for: Discovery and validation of genomic regions associated with resistance to maize lethal necrosis in four biparental populations
Source: Mol Breed. 2018 May 10;38(5):66. doi: 10.1007/s11032-018-0829-7 (PMC5945787; doi:10.1007/s11032-018-0829-7)
Supplement: Supplementary file 1 — (DOCX 101 kb) [file 11032_2018_829_MOESM1_ESM.docx]

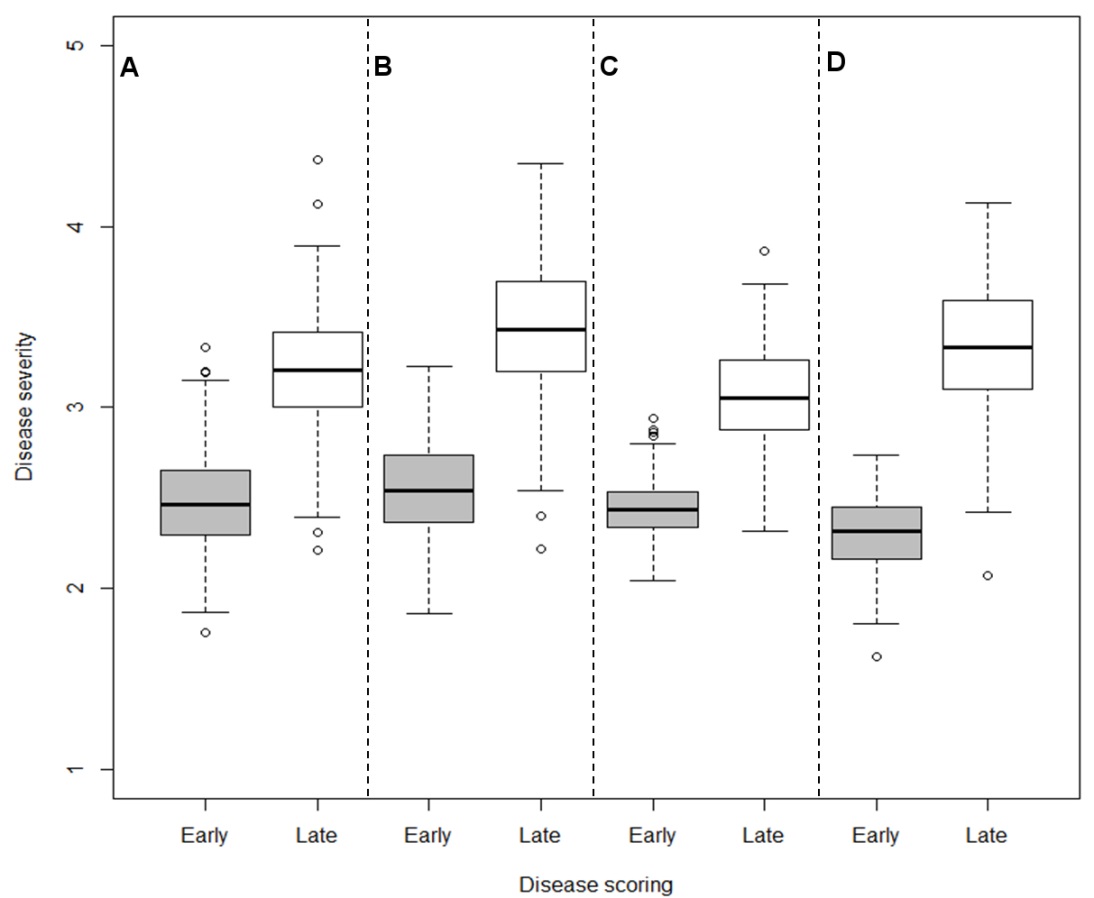


**Figure S1.** Mean distribution of MLN disease severity among the F_3_ lines derived from four different populations (A – CML543 x LaPostaSeqC7-F71; B - CML444 x CML543; C – CML539 x CML444; D - Mo37 x CML144) evaluated for MLN-early (Early) and MLN-late (Late) in three different locations.
