# Supplementary material for: Discovery and validation of genomic regions associated with resistance to maize lethal necrosis in four biparental populations
Source: Mol Breed. 2018 May 10;38(5):66. doi: 10.1007/s11032-018-0829-7 (PMC5945787; doi:10.1007/s11032-018-0829-7)
Supplement: Supplementary file 2 — (DOCX 137 kb) [file 11032_2018_829_MOESM2_ESM.docx]

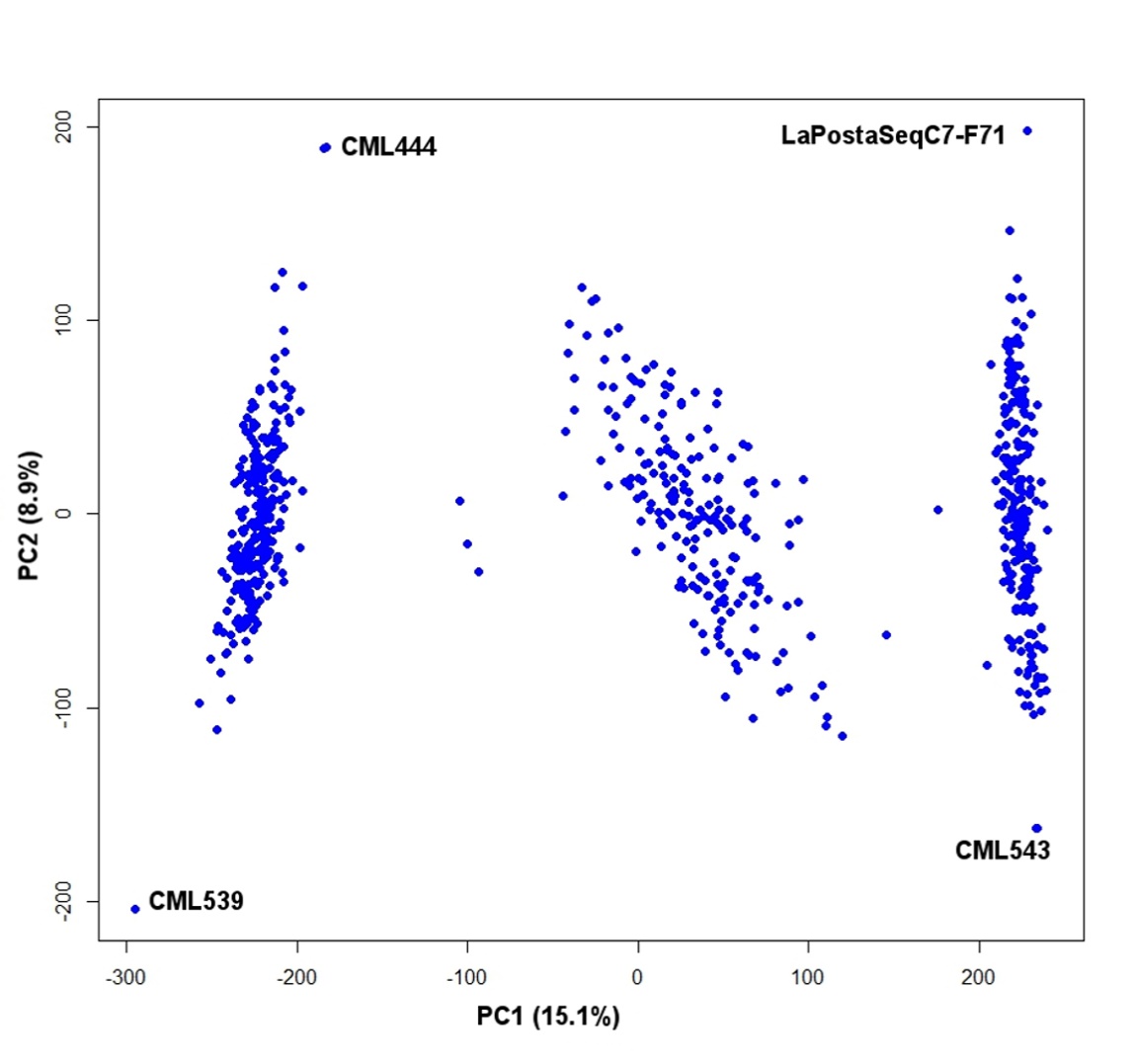


**Figure S2.** Principal component analysis of the four parents and their 689 testcross progenies. Percentages in parentheses refer to the proportion of variance explained by each principal component
