# Supplementary material for: Discovery and validation of genomic regions associated with resistance to maize lethal necrosis in four biparental populations
Source: Mol Breed. 2018 May 10;38(5):66. doi: 10.1007/s11032-018-0829-7 (PMC5945787; doi:10.1007/s11032-018-0829-7)
Supplement: Supplementary file 3 — (DOCX 1.73 mb) [file 11032_2018_829_MOESM3_ESM.docx]

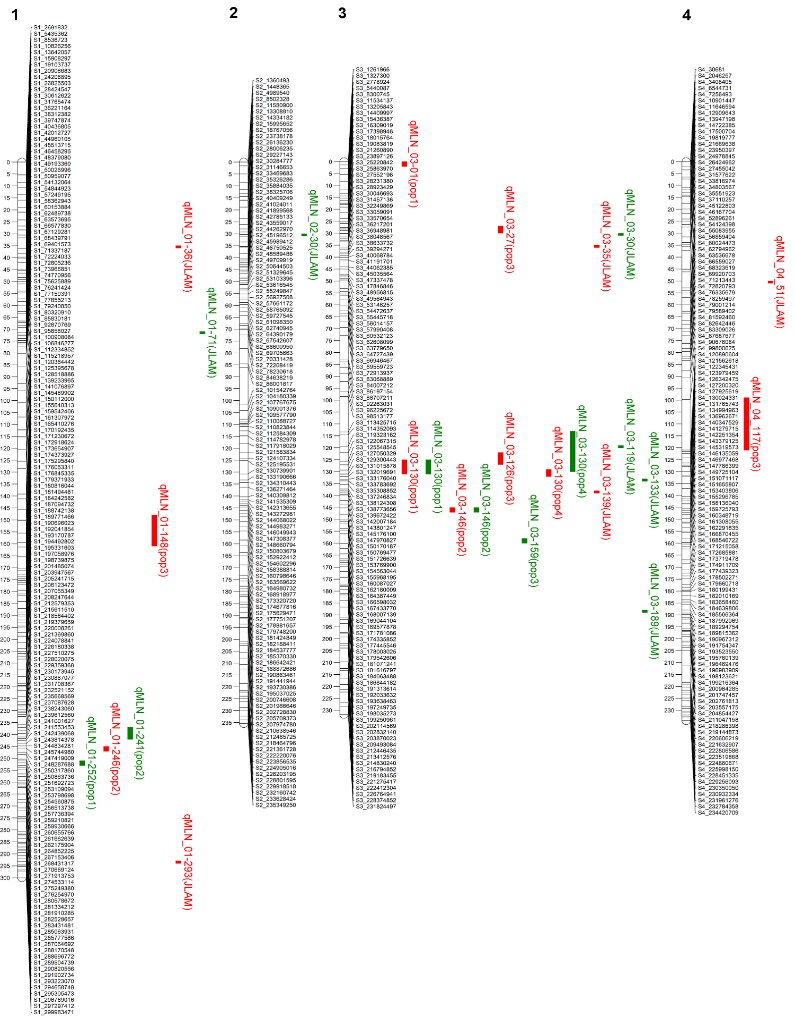

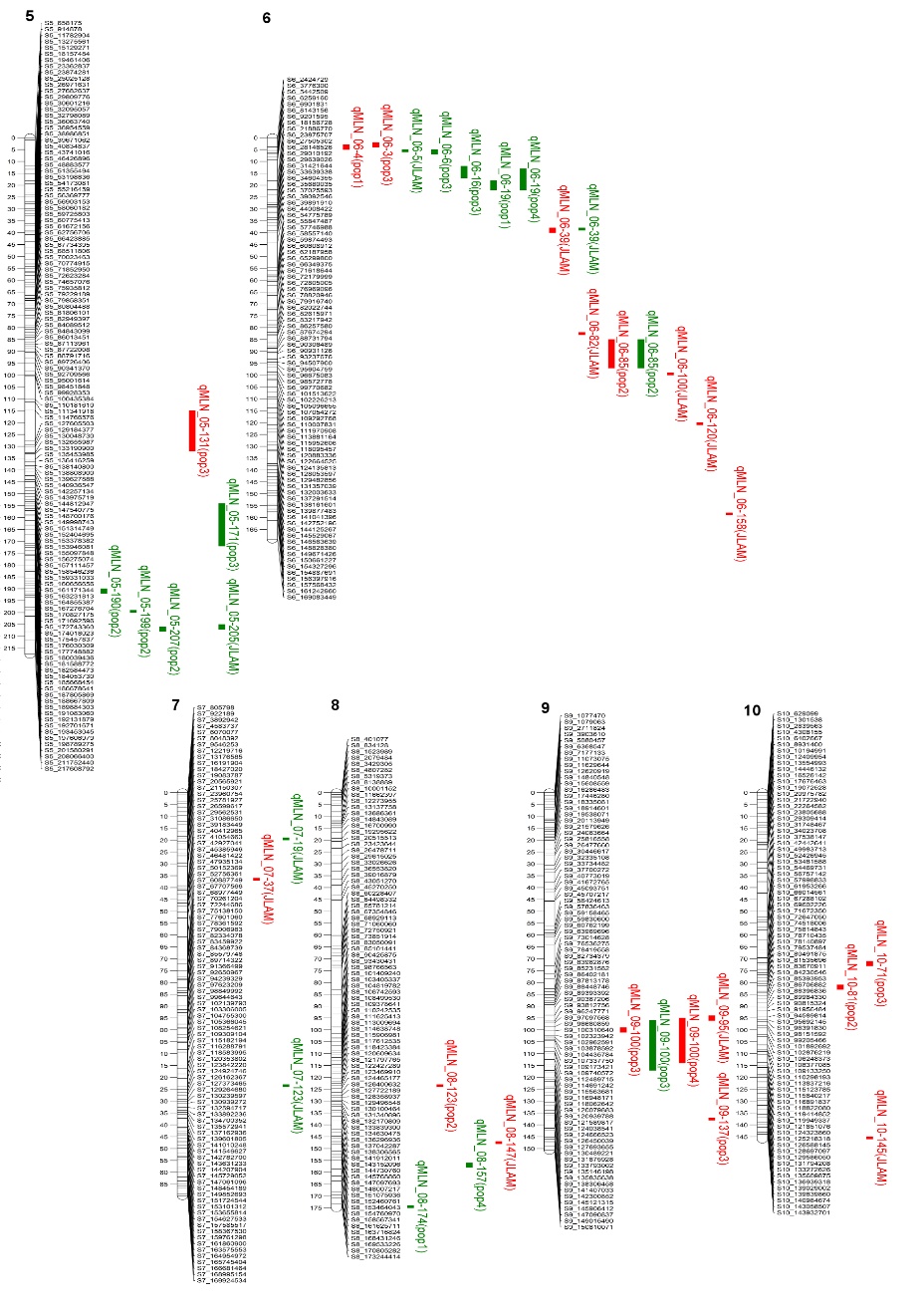


**Figure S3.** Integrated physical map and positioning of the QTL detected in four individual populations and joint linkage association mapping. The number in the left side of the map are the distance in Mbp. The labeling in the right side are the name of the SNPs. QTL name composed by the trait code followed by the chromosome number in which the QTL was mapped and a physical position of the QTL. QTL detected for MLN-early and MLN-late were marked with red and green color, respectively. Pop1 – CML543 x LaPostaSeqC7-F71; Pop2 - CML444 x CML543; Pop3 – CML539 x CML444; Pop4 - Mo37 x CML144; JLAM – Joint linkage association mapping
