# Supplementary material for: Discovery and validation of genomic regions associated with resistance to maize lethal necrosis in four biparental populations
Source: Mol Breed. 2018 May 10;38(5):66. doi: 10.1007/s11032-018-0829-7 (PMC5945787; doi:10.1007/s11032-018-0829-7)
Supplement: Supplementary file 4 — (DOCX 12.6 kb) [file 11032_2018_829_MOESM4_ESM.docx]

**Table S1**. Summary of the linkage groups constructed for four F_3_ mapping populations.

| **Population** | **No of progenies** | **No of SNPs** | **Map length** | **Avg distance (cM)** |
| --- | --- | --- | --- | --- |
| CML543 X LaPostaSeqC7-F71 | 229 | 480 | 2632.3 | 5.50 |
| CML543 X CML444 | 203 | 816 | 3696.8 | 4.51 |
| CML539 X CML444 | 260 | 378 | 2250.2 | 6.10 |
| Mo37 X CML144 | 124 | 248 | 1905.4 | 7.68 |
