# Supplementary material for: Discovery and validation of genomic regions associated with resistance to maize lethal necrosis in four biparental populations
Source: Mol Breed. 2018 May 10;38(5):66. doi: 10.1007/s11032-018-0829-7 (PMC5945787; doi:10.1007/s11032-018-0829-7)
Supplement: Supplementary file 5 — (DOCX 17.4 kb) [file 11032_2018_829_MOESM5_ESM.docx]

**Table S2:** List of candidate genes associated with significant SNPs/QTL for MLN-early and MLN-late in four different biparental populations and JLAM

| **Trait** | **QTL name^a^** | **Candidate gene** | **Annotation** |
| --- | --- | --- | --- |
| **CML543 x LaPostaSeqC7-F71** | | | |
| MLN-early | *qMLN_03-01* | GRMZM5G828179 | Zinc ion binding |
|  | *qMLN_03-130* | GRMZM2G341723 | Oxidation reduction, iron ion binding |
|  | *qMLN_06-04* | GRMZM2G030325 | Regulation of transcription |
| MLN-late | *qMLN_01-252* | GRMZM2G173425 | Zinc finger protein LSD2 |
|  | *qMLN_03-130* | GRMZM2G341723 | Oxidation reduction, iron ion binding |
|  | *qMLN_06-19* | GRMZM2G700866 | Protein serine/threonine kinase activity |
|  | *qMLN_08-174* | GRMZM5G874163 | Regulation of transcription, DNA binding |
| **CML444 x CML543** | | | |
| MLN-early | *qMLN_01-246* | GRMZM2G011912 | Putative uncharacterized protein |
|  | *qMLN_03-146* | GRMZM2G309512 | Protein serine/threonine kinase activity |
|  | *qMLN_06-85* | GRMZM2G108798 | homoiothermy, ice binding, response to freezing |
|  | *qMLN_08-123* | GRMZM2G096115 | Protein serine/threonine kinase activity, signal transduction |
|  | *qMLN_10-81* | GRMZM2G325907 | Myb-like DNA-binding domain containing protein |
| MLN-late | *qMLN_01-241* | GRMZM2G124434 | Catalytic activity |
|  | *qMLN_03-146* | GRMZM2G309512 | Protein serine/threonine kinase activity |
|  | *qMLN_05-190* | GRMZM2G156599 | transmembrane transport, Iron-phytosiderophore transporter |
|  | *qMLN_05-199* | GRMZM2G103357 | protein transport, integral to membrane |
|  | *qMLN_05-207* | GRMZM2G105644 | Oxidoreductase activity acting on the CH-CH group of donors NAD or NADP as acceptor |
|  | *qMLN_06-85* | GRMZM2G108798 | homoiothermy, ice binding, response to freezing |
| **CML539 x CML444** | | | |
| MLN-early | *qMLN_01-148* | GRMZM2G382557 | Cell wall modification |
|  | *qMLN_03-27* | GRMZM2G018664 | Oxidoreductase activity |
|  | *qMLN_03-126* | GRMZM2G151720 | Putative uncharacterized protein |
|  | *qMLN_04-117* | GRMZM2G423663 | Protein binding |
|  | *qMLN_05-131* | GRMZM2G452633 | Putative uncharacterized protein |
|  | *qMLN_06-03* | GRMZM2G374827 | homoiothermy, ice binding, response to freezing |
|  | *qMLN_09-100* | GRMZM2G034069 | Cellular metabolic process, oxidoreductase activity |
|  | *qMLN_10-71* | GRMZM2G055070 | Regulation of transcription DNA-dependent |
| MLN-late | *qMLN_03-159* | GRMZM5G891247 | response to freezing, enzyme inhibitor activity |
|  | *qMLN_05-171* | GRMZM5G839969 | transcription factor activity |
|  | *qMLN_06-06* | GRMZM2G054946 | Nucleoside-triphosphatase activity, defense response, apoptosis, proteolysis |
|  | *qMLN_06-16* | GRMZM2G001924 | Protein serine/threonine kinase activity |
|  | *qMLN_09-100* | GRMZM2G034069 | Cellular metabolic process, oxidoreductase activity |
| **Mo37 x CML144** | | | |
| MLN-early | *qMLN_03-130* | GRMZM2G341723 | oxidation reduction, iron ion binding |
|  | *qMLN_09-100* | GRMZM2G034069 | Cellular metabolic process, oxidoreductase activity |
| MLN-late | *qMLN_03-129* | GRMZM2G112074 | nucleotide binding, DNA replication |
|  | *qMLN_06-19* | GRMZM2G700866 | Protein serine/threonine kinase activity |
|  | *qMLN_08-157* | GRMZM2G176301 | C-4 methylsterol oxidase, ion binding |
| **JLAM** | | | |
| MLN_early | *qMLN_01-36* | GRMZM2G127123 | Hydrolase activity hydrolyzing O-glycosyl compounds |
|  | *qMLN_01-293* | GRMZM2G155317 | Putative uncharacterized protein |
|  | *qMLN_03-56* | GRMZM2G409309 | No significant hits |
|  | *qMLN_03-139* | GRMZM2G101221 | Response to oxidative stress, peroxidase activity |
|  | *qMLN_04-05* | GRMZM2G044152 | Hypothetical protein |
|  | *qMLN_06-21* | GRMZM2G050561 | Translation, aminoacyl-tRNA ligase activity, ATP binding |
|  | *qMLN_06-39* | GRMZM2G066326 | Cysteine-type endopeptidase activity |
|  | *qMLN_06-82* | GRMZM2G000376 | Putative uncharacterized protein |
|  | *qMLN_06-100* | GRMZM2G071582 | Zinc ion binding, ARF GTPase activator activity |
|  | *qMLN_06-120* | GRMZM2G036564 | Embryogenesis transmembrane protein |
|  | *qMLN_06-158* | GRMZM2G435475 | Regulation of transcription, zinc ion binding |
|  | *qMLN_07-037* | GRMZM2G028041 | Homeobox protein rough sheath, transcription factor |
|  | *qMLN_08-147* | GRMZM2G171279 | Transmembrane transport, ion channel activity |
|  | *qMLN_09-95* | GRMZM2G117935 | Hydrogen ion transporting ATP synthase activity |
|  | *qMLN_09-137* | GRMZM2G083222 | Hydrolase activity hydrolyzing O-glycosyl compounds |
|  | *qMLN_10-145* | GRMZM2G421033 | Dehydration responsive element binding protein |
|  | | | |
| MLN-late | *qMLN_01-07* | GRMZM2G090568 | Response to oxidative stress, iron ion binding |
|  | *qMLN_02-30* | GRMZM2G111954 | Oxidoreductase activity |
|  | *qMLN_03-119* | GRMZM2G390345 | Protein serine/threonine kinase activity |
|  | *qMLN_03-133* | GRMZM5G804881 | Oxidation reduction, NADPH binding |
|  | *qMLN_03-189* | GRMZM2G306143 | Serine-type endopeptidase activity |
|  | *qMLN_05-205* | GRMZM2G090609 | MAIZE Caleosin related protein |
|  | *qMLN_06-05* | GRMZM2G054946 | Nucleoside-triphosphatase activity, defense response, apoptosis, proteolysis |
|  | *qMLN_06-39* | GRMZM2G066326 | Cysteine-type endopeptidase activity |
|  | *qMLN_07-19* | GRMZM2G111216 | Homoiothermy, ice binding, response to freezing |
|  | *qMLN_07-123* | GRMZM5G847216 | Putative uncharacterized protein |

*MLN-early= MLN score 21 days after 1st post inoculation; MLN-late = MLN score 42 days after 1st post inoculation; ^a^ QTL name composed by the trait code followed by the chromosome number in which the QTL was mapped and a physical position of the QTL
